# SLC39A1 contribute to malignant progression and have clinical prognostic impact in gliomas

**DOI:** 10.1186/s12935-020-01675-0

**Published:** 2020-11-27

**Authors:** Peng Wang, Jingjing Zhang, Shuai He, Boan Xiao, Xiaobin Peng

**Affiliations:** 1 The Fifth Affiliated Hospital of Southern Medical University, Guangzhou, 510900 China; 2 Zhujiang Hospital, Southern Medical University, Guangzhou, 510282 China

**Keywords:** Glioma, SLC39A1, Malignant progression, Clinical prognostic impact, MMP2\MMP9, Immune infiltration

## Abstract

**Background:**

Gliomas are one of the most common primary tumors of the central nervous system, and have an unfavorable prognosis. SLC39A1 is a zinc ion transport protein which inhibits the progression of prostate cancer. By studying the role and mechanism of SLC39A1 in the progression of gliomas, perhaps a new therapeutic target can be provided for their treatment.

**Method:**

The TCGA, CCGA, GSE16011, GSE44971 and GSE11260 data sets were employed to evaluate the expression level of SLC39A1 in paracancerous and glioma tissues. In addition, Kaplan–Meier analysis, Cox analysis, and the ESTIMATE and CIBERSORT algorithms were used to analyze its prognostic value and immune infiltration correlation. A CCK-8 and flow cytometer were used to measure the effects of SLC39A1 on U87 cell proliferation or apoptosis; RT-qPCR and western blot were used to detect its effects on the expression of MMP2\MMP9.

**Results:**

SLC39A1 has up-regulated expression in glioma tissues. High SLC39A1 expression predicted significantly worse survival. Univariate and multivariate analysis show that SLC39A1 independently indicated poor prognosis in patients with gliomas. The expression of SLC39A1 is significantly correlated with clinical pathological parameters such as Grade, IDH mutation status, and 1p19q codeletion status. In vitro experimental results show that SLC39A1 promotes proliferation of glioma cells, inhibits their apoptosis, and promotes expression of MMP2\MMP9. In addition, it may affect infiltration of immune cells into the glioma microenvironment.

**Conclusion:**

SLC39A1 may serve as a new prognostic biomarker and potential target for treatment of gliomas.

## Background

Gliomas are one of the most common and aggressive primary tumors in the central nervous system (CNS). Their incidence accounts for 40% to 50% of the total incidence of intracranial tumors. They are highly malignant and result in a short survival time. In particular, the median survival time for patients suffering from glioblastoma multiforme (GBM), one of the most well-known malignant tumors in humans [1–3], is only 12-15 months. The average one-year survival rate is about 46%, while the 5-year survival rate is only 3%. The main clinical treatments for gliomas are currently surgery, radiotherapy and chemotherapy, but they usually fail to achieve the desired results, and the clinical prognosis is extremely poor [4, 5]. Therefore, further research on the occurrence and development of gliomas, and new molecular targets for their diagnosis and treatment, is of great practical importance.

SLC39A proteins are ZIP metal ion transport proteins, and are widely found in a variety of tissues [6]. They are mainly distributed on the plasma membrane to absorb Zn^2+^ and increase the availability of cytoplasmic Zn^2+^ [7]. The human genome includes 14 SLC39A proteins (SLC39A1-14). Abnormal expression of the SLC39A family of proteins leads to cell Zn^2+^ metabolism disorders, which has been proved to be related to cancers such as pancreatic cancer, cervical cancer and lung cancer. This may be due to the fact that Zn^2+^ is a cofactor of matrix metalloproteinases (MMPs), carbonic anhydrase, and other enzymes, while MMPs and carbonic anhydrase are involved in tumor proliferation, migration and infiltration [8]. For instance, SLC39A6 can enhance the aggressive phenotype by inducing human pancreatic cancer cell epithelial-mesenchymal transition [9]; SLC39A7 silencing significantly up-regulates expression of Bax and E-cadherin, down-regulates expression of Bcl-2 and MMP2, and inhibits cell proliferation, migration and invasion of cervical cancer [10]; SLC39A4 increases lung cancer cell epithelial-mesenchymal transition and inhibits the sensitivity of cisplatin to cancer cells, promoting lung cancer cell proliferation and invasion [11]. SLC39A1, coded hZip1/ZIRTL, is the first member of SLC39A. It is located in the plasma membrane and has zinc absorption activity [6, 12]. Although it is widely distributed across various tissue cells, its role in tumors has only been confirmed in prostate cancer. Studies have shown that the level of zinc in normal human prostates is about 15 times higher than in other tissues [13]. Low expression of SLC39A1 decreases the level of Zn^2+^ in prostate cancer tissue, thereby reducing the level of citrate, and ultimately resulting in the malignant progression of prostate cancer [14, 15]. Therefore, SLC39A1 may play an important role in tumor progression. To this point, however, there is little information about its role in central nervous system tumors. Its expression and clinical significance in gliomas are still unclear.

In this study, we used TCGA, CGGA, and GEO databases to comprehensively analyze the expression of SLC39A1 and its role in the prognosis of glioma patients. In the meantime, we used a CCK-8, flow cytometer, RT-qPCR, western blot, and other technologies to analyze the correlation between SLC39A1 and glioma proliferation and apoptosis in vitro, and the expression of invasion-related MMP2\MMP9 proteins. In addition, we also used the ESTIMATE and CIBERSORT algorithms to evaluate the correlation between SLC39A1 and TME immune cell infiltration. The findings in this report reveal the important role and mechanism of SLC39A1 in the progression of gliomas, and this report also analyzes the correlation between SLC39A1 and immune cell infiltration in the glioma microenvironment.

## Methods and methods

### Data download and processing

Gene expression and corresponding clinical data was downloaded from the TCGA, CGGA and GEO databases. TCGA is an open database containing expression and clinical data for 29 different tumors, from which we downloaded the data on gliomas. GSE16011, GSE44971, and GSE11260 are public data sets in the GEO database, and respectively contain 284, 58, and 70 glioma or paracancerous samples. CGGA refers to the Chinese Glioma Genome Atlas, from which we downloaded two datasets B and C, which respectively contain 693 and 325 mRNA-seq samples, and we used the “limma” and “sva” packages in R to correct and combine the two sets of gene expression data in batches.

### Bioinformatic analysis

The TCGA, GSE16011, GSE44971 and GSE11260 data sets were used to analyze the expression differences of SLC39A1 in cancer and paracancerous tissues, while TCGA and CGGA were used to analyze its survival, prognosis and clinical correlations. The “survival” and “survminer” packages in R were used for survival and cox analysis; “beeswarm” was used for clinical correlation analysis.

### Gene enrichment analysis

Biological processes related to SLC39A1 expression were detected through GO and KEGG pathway analysis. The richGO function of R was used for GO analysis, and the enrichKEGG function for KEGG pathway analysis. P values indicate the significance of related functions or pathways (the recommended critical value is 0.05).

### Cell cultures

U87MG cells were purchased from Land Biotechnology (Guangzhou, China). 10% FBS was added in DMEM, then they were placed in a 37 °C, 5% CO2 incubator for culturing.

### Interference and over-expression

For SiRNA screening and plasmid construction, refer to the method described in study [16]. SiRNA and over-expression plasmids were synthesized by Land Biotechnology (Guangzhou, China). Refer to the instructions for use of Lipofectamine 2000 (Invitrogen, USA) to transfect siRNA and over-expression plasmid into U87 cells.

### RT-qPCR and western blot

RT-qPCR and western blot analysis were performed using the method described in study [16]. Briefly, when performing RT-qPCR analysis, the TaKaRa (Japan) reverse transcription kit was first used to synthesize SS1 cDNA, then t-he TaKaRa (Japan) SYBR^®^Premix Ex Taq™ kit and real-time quantitative PCR system were used for real-time amplification reaction monitoring. GAPDH was used as a control, and the mRNA expression results were corrected. The following primer sequences were utilized: SLC39A1 forward, 5′-GAACAAGAGATGGTCAAGTC-3′ and reverse, 5′-ATGTGAGCCTGTCCTTATG-3′; MMP2 forward, 5′-CTCGGTAGGGACATGCTAAGTAGAG-3′ and reverse, 5′-CCTCTGGAGGTTCGACGTGA-3′; MMP9 forward 5′-TGACAGCGACAAGAAGTG-3′ and r-everse 5′-TGACAGCGACAAGAAGTG-3′;and GAPDH forward, 5′-GAAGGTGAAGGTCGGAGTC-3′ and reverse, 5′-GAAGATGGTGATGGGATTTC-3′.

The western blot undergoes processes including total protein extraction, preliminary quantification of protein samples, SDS-PAGE electrophoresis, protein transfer, and immunoblotting. GAPDH (Aksomics, China) as loading control used the following antibodies: Anti-SLC39A1 antibody (ab105416) (Abcam, China), Anti-MMP2 antibody (ab97779) (Abcam, China), Anti-MMP9 antibody (ab38898) (Abcam, China), and Goat Anti-Rabbit IgG H&L (HRP) (ab205718) (Abcam, China), etc.

### CCK-8 assay

A Cell Counting Kit-8 (Invitrogen, USA) was used to detect cell viability according to its instructions. U87 cells from different groups (Con, overexpression, NC, siRNA and si-NC) were inoculated in 96 pore plates, each group with 3 replicated pores (1 × 104 cells/pore) and placed in a 37 °C, 5% CO2 incubator for culturing. After 3 days, 10 µL of CCK-8 solution was added to each pore. After 4 h, a microplate reader (multiscan MK3, Thermo Fisher Scientific) was used for measurement (wavelength 450 nm).

### Apoptosis test

The apoptosis rate was measured using an Annexin V-FITC Apoptosis Detection Kit (Beyotime, China). Following the instructions, the collected cells were first digested with pancreatic enzymes, and then centrifuged to remove the supernatant and washed with PBS 3–4 times. Finally, Annexin V-FITC and Bingding Buffer were added for measurement. A BD (US) flow cytometer to measure and analyze the apoptosis rate.

### Immune infiltration analysis

The ESTIMATE algorithm in the R estimate package was used to estimate the ratio of the immune matrix components of each sample in the TME, which is presented in the form of three scores: ImmuneScore, StromalScore and ESTIMATEScore. Based on this, TumorPurity was then estimated, and the correlation between them and the expression of SLC39A1 was calculated, thus evaluating the effect of SLC39A1 expression on immune infiltration. In addition, the CIBERSORT algorithm was used to analyze the proportions of tumor infiltration immune subgroups and evaluate their correlation with SLC39A1 expression, further verifying the effect of SLC39A1 expression on immune cell infiltration in the tumor microenvironment.

### Statistical analysis

Quantitative data are presented as mean ± SD. the Significant differences among groups was estimated by Student’s test and one-way ANOVA. Kaplan–Meier survival analysis was used to assess The prognostic value of SLC39A1. Univariate and multivariate Cox regression analysis were performed to test independent prognostic factors in R 3.6.2. P < 0.05 was considered statistically significant.

## Results

### SLC39A1 expression in gliomas

**Fig. 1:**
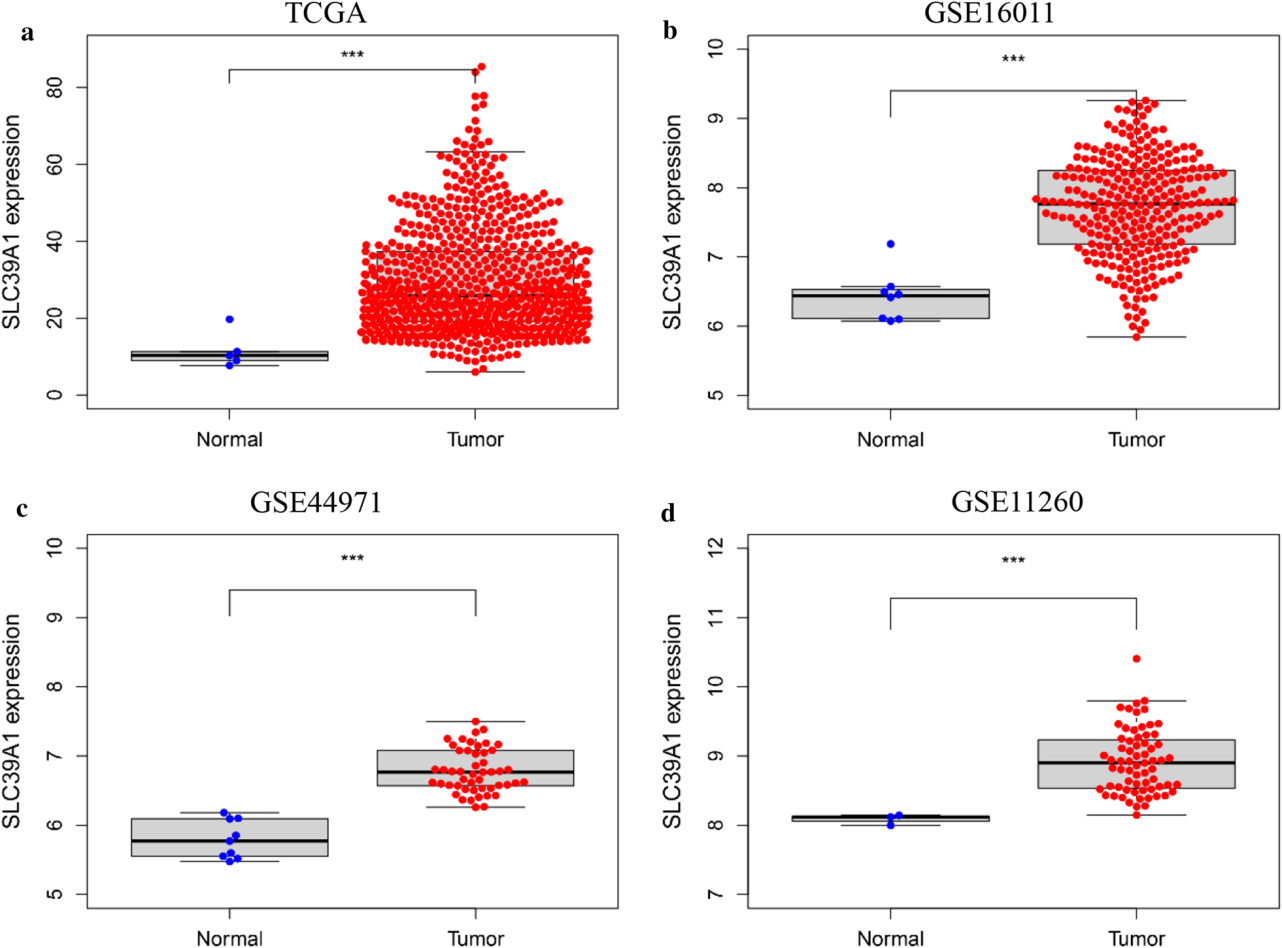
Expression of SLC39A1 in normal and glioma tissues. SLC39A1 expression in normal and glioma tissues from **a** TCGA datasets (n = 705) , **b** GSE16011 datasets (n = 284), **c** GSE44971 datasets (n = 58), **d** GSE11260 datasets (n = 70). TCGA, The Cancer Genome Atlas. ***P < 0.001 indicates a significant difference

The TCGA database was used to analyze the expression of SLC39A1 in gliomas. The results showed that the expression of SLC39A1 was significantly higher in gliomas than in paracancerous tissues (Fig. 1a). In order to verify this finding, we downloaded three data sets from the GEO database, GSE16011, GSE44971, and GSE11260, and analyzed SLC39A1 expression among them. The results showed that in the three data sets, SLC39A1 expression in gliomas was significantly higher than in the paracancerous tissues (Fig. 1b, c, d), consistent with the analysis results from the TCGA data set.

### High expression of SLC39A1 is associated with poor glioma prognosis

**Fig. 2:**
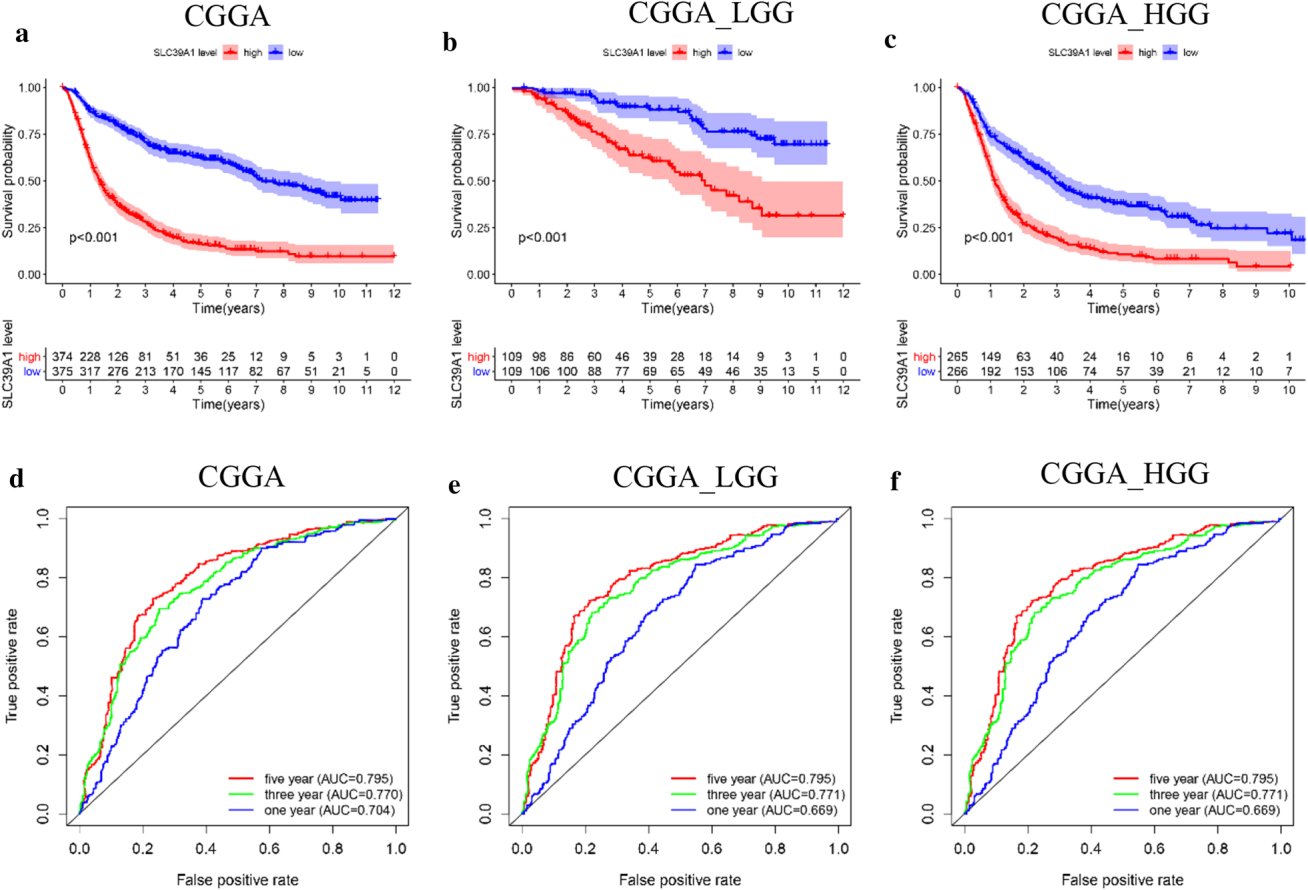
SLC39A1 expression is associated with prognosis in patients with glioma. (**a, b, c**) Kaplan–Meier analyses of patients with glioma (**a**), LGG (**b**) or HGG (**c**) in different expression level of SLC39A1. The red curve represents high expression and the blue curve represents low expression. P < 0.05 indicates a significant difference. **d, e, f** ROC (Receiver operator characteristic curve) analysis of SLC39A1 in patients with glioma (**d**), LGG (**e**) or HGG (**f**). *AUC* area under the curve

We employed the TCGA and CGGA databases to study the role of SLC39A1 in the prognosis of glioma patients. Kaplan–Meier analysis results showed that in the CGGA dataset, patients with high SLC39A1 expression had a shorter overall survival time (Fig. 2a). Due to the significant difference between HGG and LGG, we further analyzed the prognostic value of SLC39A1 in LGG and HGG patients. The patients were divided into two groups based on SLC39A1expression level. We found that in the high expression group, the prognosis of both LGG or HGG patients was poor (Fig. 2b, c). Receiver operating characteristic (ROC) curve analysis showed that SLC39A1 is a predictor of 1-year, 3-year, and 5-year survival (Fig. 2d, e, f). Similar analysis results were also obtained in the TCGA database (Additional file 1: Fig. S1).

### SLC39A1 independently indicated poor prognosis in patients with gliomas

**Fig. 3:**
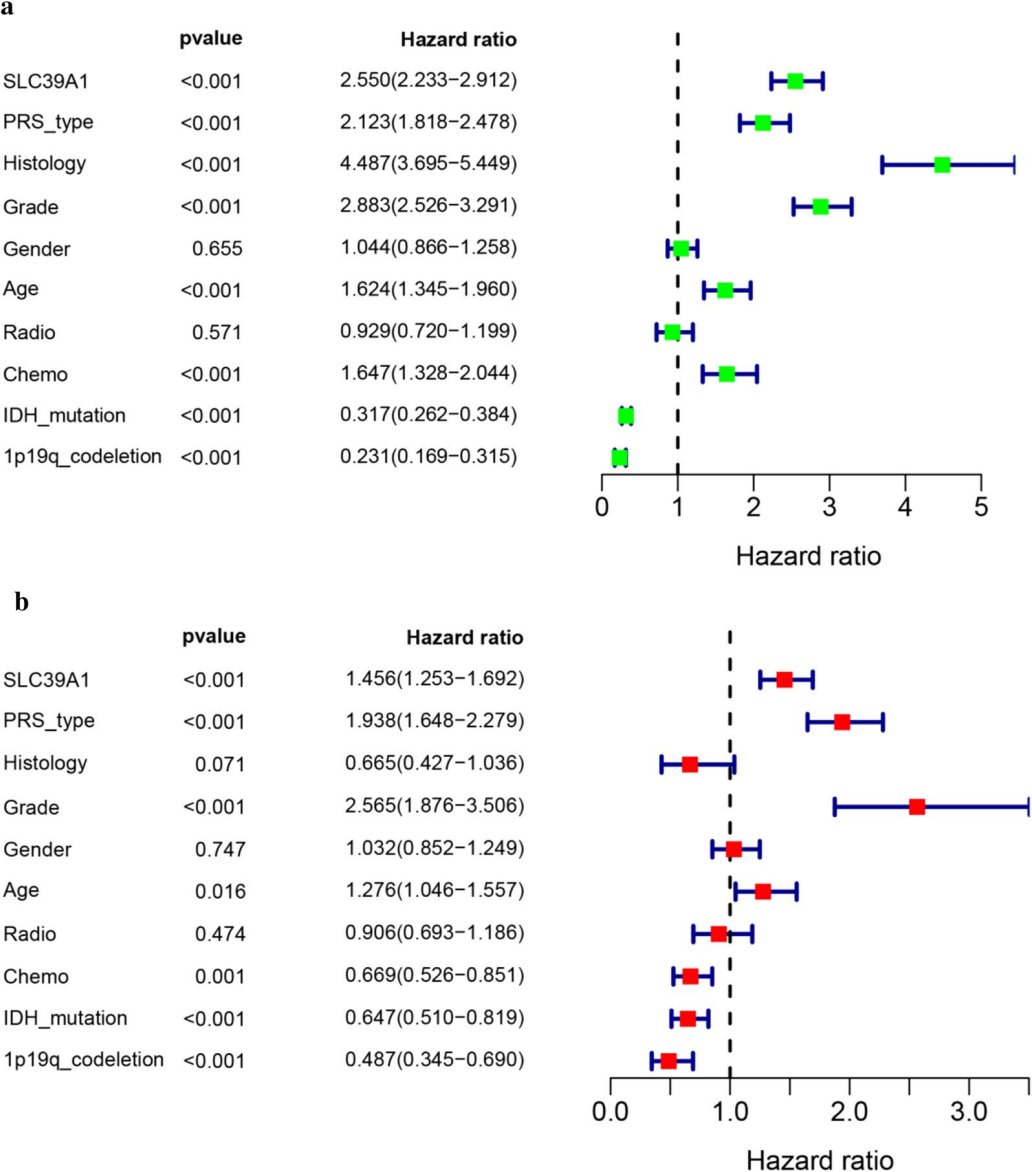
Cox analyses of SLC39A1 using CGGA database. **a** Univariate analysis of SLC39A1. **b** Multivariate analysis of SLC39A1. P < 0.05 indicates a significant difference

Since the prognosis of glioma patients is affected by a variety of clinicopathological factors, in order to further evaluate the prognostic value of SLC39A1 in glioma patients in the CGGA database, we used cox regression analysis. Univariate cox analysis showed that SLC39A1, PRS_type, Histology and Grade were high-risk factors, while IDH_mutation and 1p19q_codeletion were low-risk factors (Fig. 3a). Multivariate cox analysis showed that SLC39A1 might be an independent factor affecting the prognosis of patients with glioma (HR = 1.456; 95% CI 1.253–1.692; P < 0.001) (Fig. 3b).

### The relationship between SLC39A1 expression and clinicopathological parameters

**Fig. 4:**
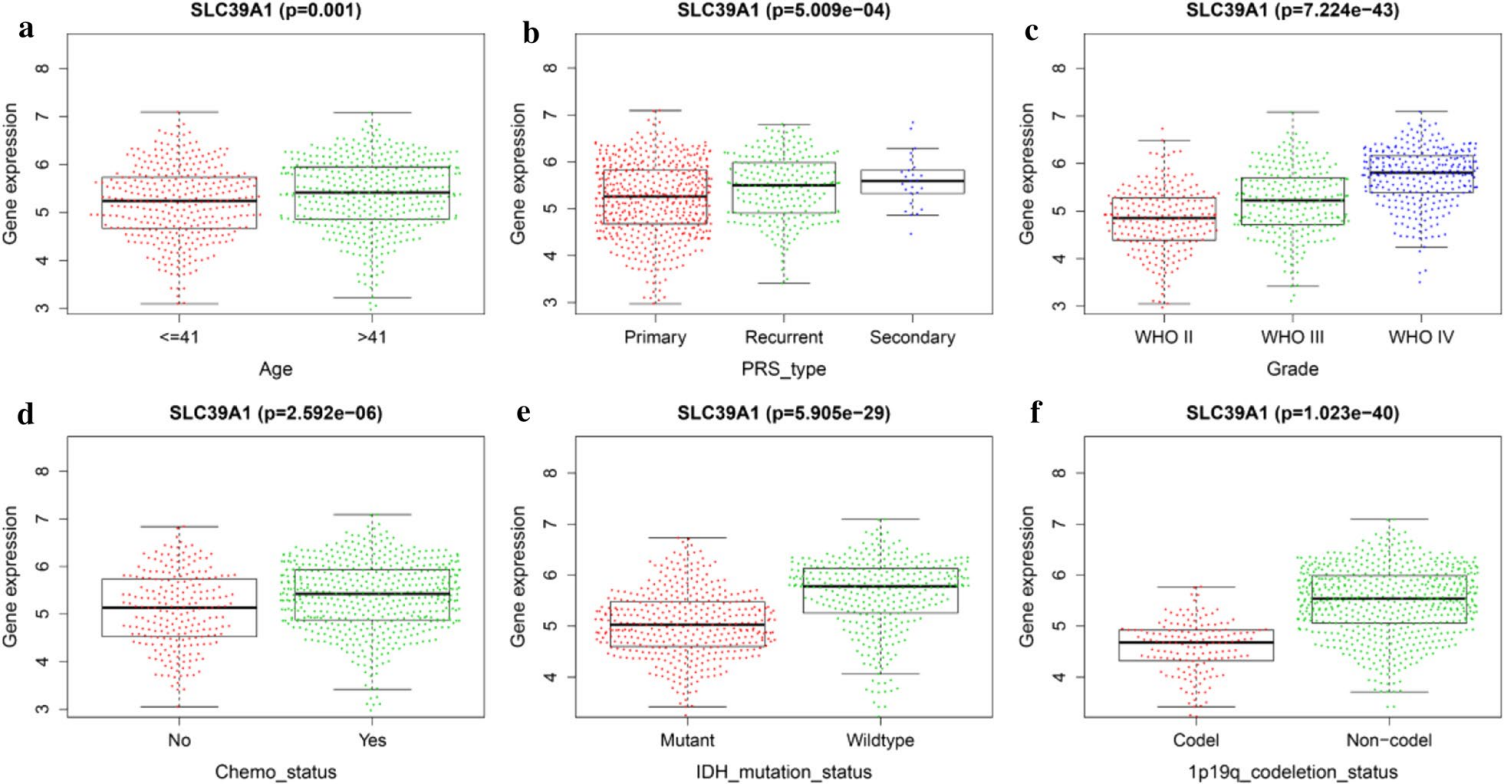
Correlation analysis between SLC39A1 expression and clinicopathological parameters using CGGA database. Differential expression of SLC39A1 was significantly related to **a** Age, **b** PRS_type, **c** Grade, **d** Chemo_status, **e** IDH mutation status and **f** 1p19q codeletion status. P < 0.05 indicates a significant difference

In order to study the relationship between SLC39A1 expression and clinicopathological parameters, we downloaded clinical data for 1018 gliomas from CGGA. Using the median level, we divided the samples into high and low SLC39A1 expression groups. Correlation analysis showed that SLC39A1 expression was significantly correlated with PRS type, histology, grade, age, chemo status, IDH mutation status, and 1p19q codeletion status (Fig. 4). In addition, the 700 glioma clinical samples downloaded from the TCGA data had similar analysis results (Additional file 2: Fig. S2).

### Gene enrichment analysis

**Fig. 5:**
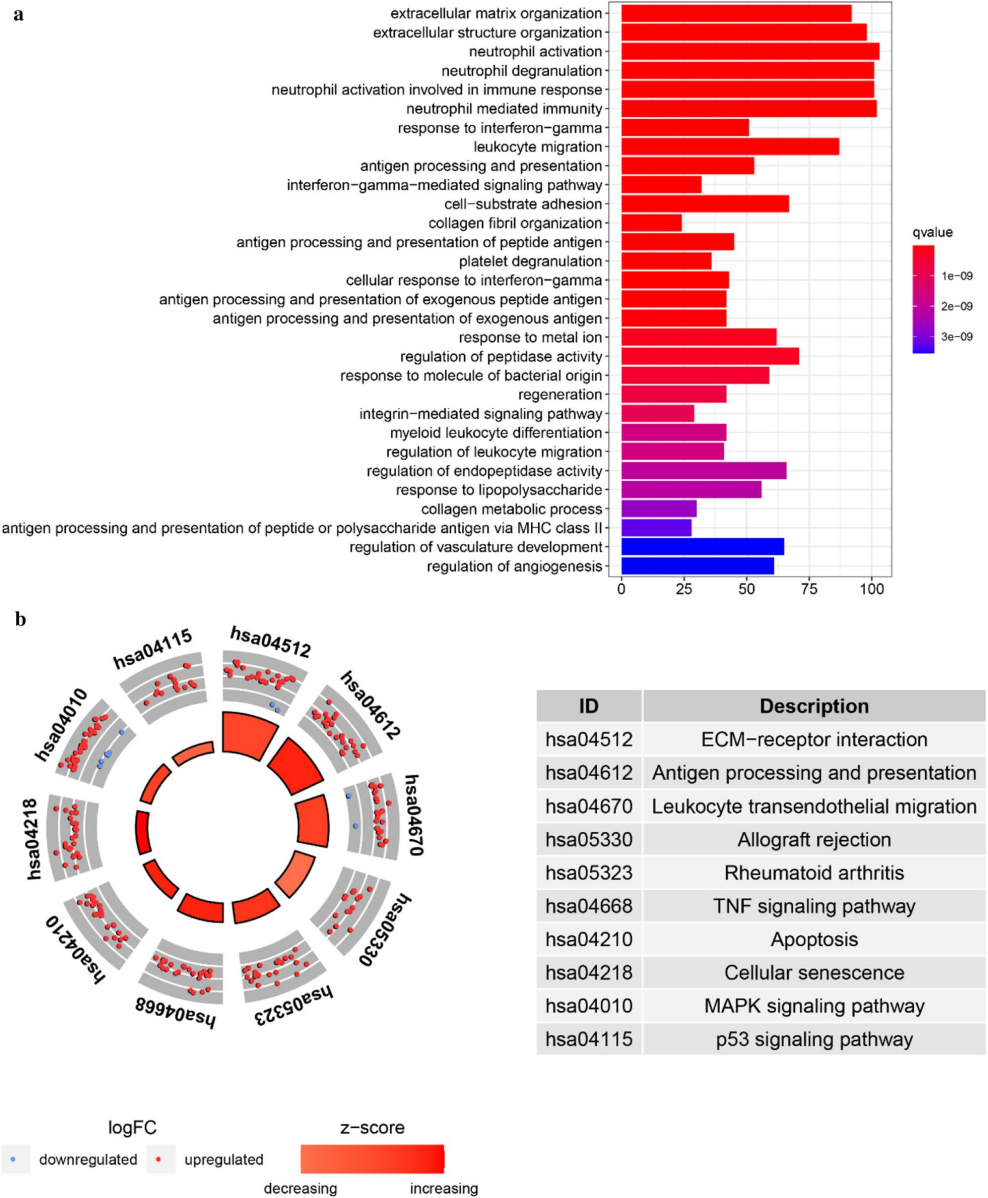
Gene enrichment analysis of SLC39A1 based on CGGA dataset. **a** Gene Ontology analysis. **b** KEGG pathway analysis

The CGGA database samples were divided into high and low expression groups using the median of SLC39A1 expression according to the results of glioma mRNA sequencing. Gene enrichment analysis was used to determine the enrichment functions and pathways of the differentially expressed genes between the two groups. GO analysis shows that the main functions in which SLC39A1 is enriched are extracellular matrix organization, extracellular structure organization, neutrophil activation, and leukocyte migration (Fig. 5a); KEGG analysis shows that the main pathways in which SLC39A1 is enriched are ECM-receptor interaction, Antigen processing and presentation, Leukocyte transendothelial migration, and the TNF signaling pathway (Fig. 5b). In addition, analysis on the TCGA dataset also obtains similar results (Additional file 3: Fig. S3).

### Role of SLC39A1 in extracellular matrix tissue of gliomas

**Fig. 6:**
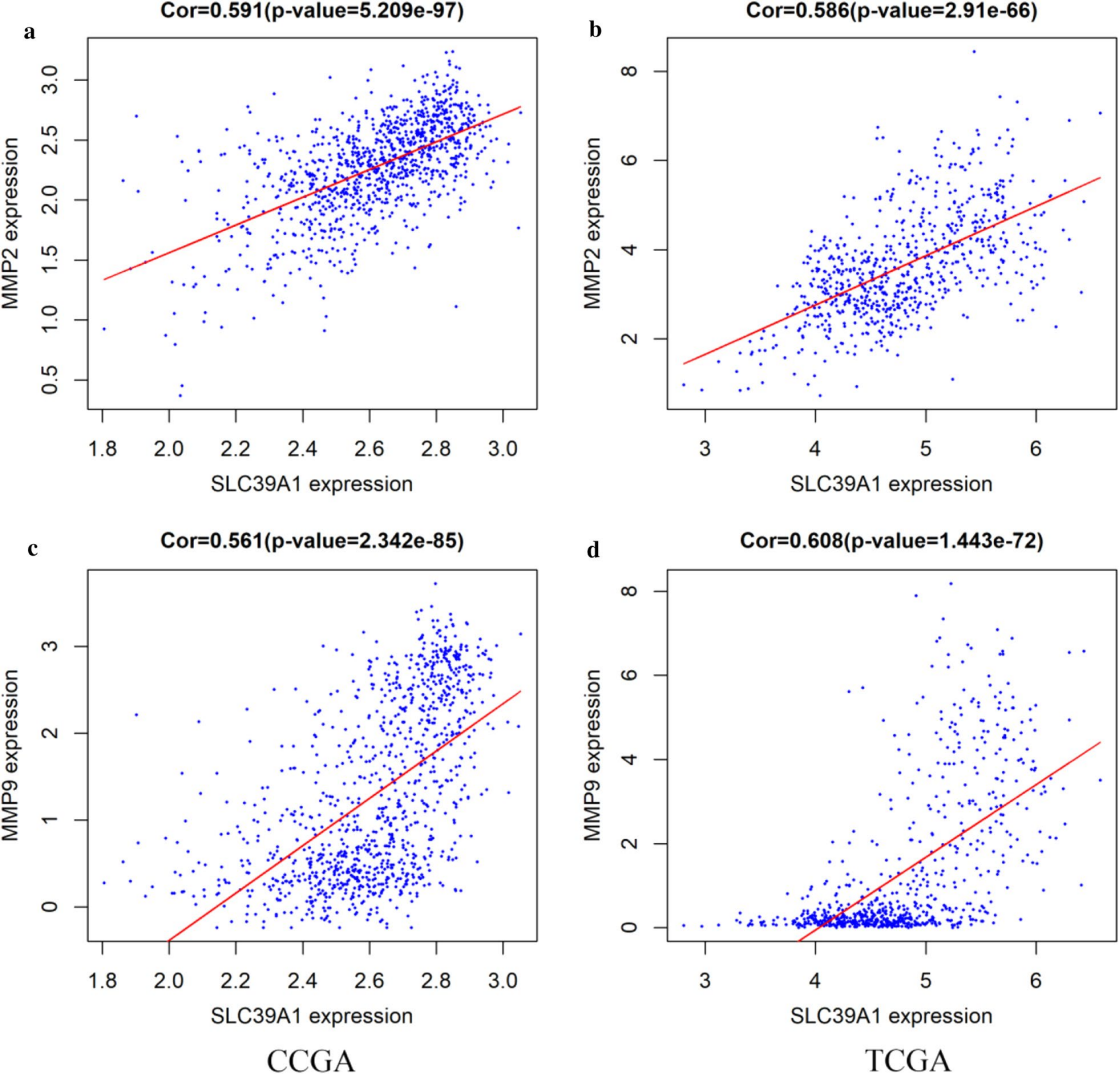
Correlation between SLC39A1 and invasion‑related markers in glioma using CGGA and TCGA database. **a, c** data from CGGA database. **b, d** data from TCGA database. P < 0.05 indicates a significant difference

Based on the gene function enrichment analysis, SLC39A1 is mainly enriched in extracellular matrix and extracellular structure tissue. This suggests that SLC39A1 may be related to the proliferation, metastasis and invasion ability of gliomas. To further analyze this result, we used the CGGA and TCGA datasets to analyze the association between SLC39A1 expression and various invasion-related proteins. The results showed that SLC39A1 was significantly positively correlated with the expression of MMP2 and MMP9 (P < 0.0001, Fig. 6).

### SLC39A1 promotes glioma progression in vitro

In order to study the role of SLC39A1 in gliomas, we stably silenced or overexpressed SLC39A1 by transfecting SLC39A1 siRNA or SLC39A1 overexpression plasmid. As shown in Fig. 8a, the expression of SLC39A1 in the siRNA group (SLC39A1 knockout group) was significantly reduced (P < 0.05), while its expression in the overexpression group (SLC39A1 overexpression group) was significantly increased, indicating a successful transfection.

**Fig. 7:**
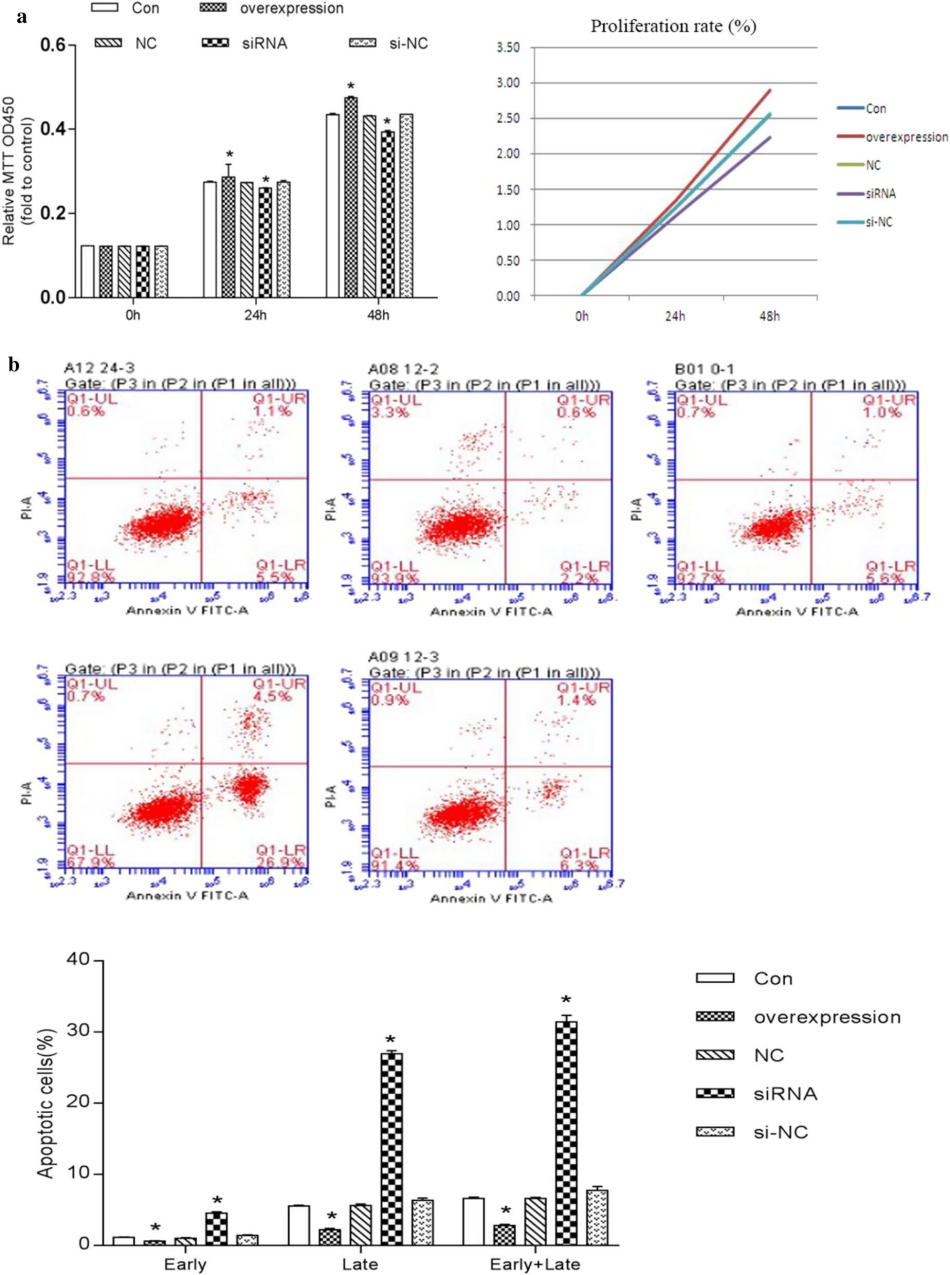
Effects of SLC39A1 on proliferation (**a**) and apoptosis (**b**) of U87 MG cells. Con, negative control group; overexpression, SLC39A1 overexpression group; NC, plasmid control group; siRNA, SLC39A1 knockdown groups; si-NC, negative control siRNA. *P<0.05

We used CCK-8 to measure the effect of SLC39A1 on the proliferation of U87 cells. The results (Fig. 7a) showed that compared with Con, cell proliferation in the overexpression group was significantly increased (P < 0.05), while that in the siRNA group was significantly decreased (P < 0.05).

We employed a flow cytometer to measure cell apoptosis. The results showed (Fig. 7b) that cell apoptosis in the overexpression group (P < 0.05) was significantly reduced compared with Con, while that in the siRNA group (P < 0.05) was significantly increased.

### SLC39A1 significantly increased the expression of MMP2 and MMP9

**Fig. 8:**
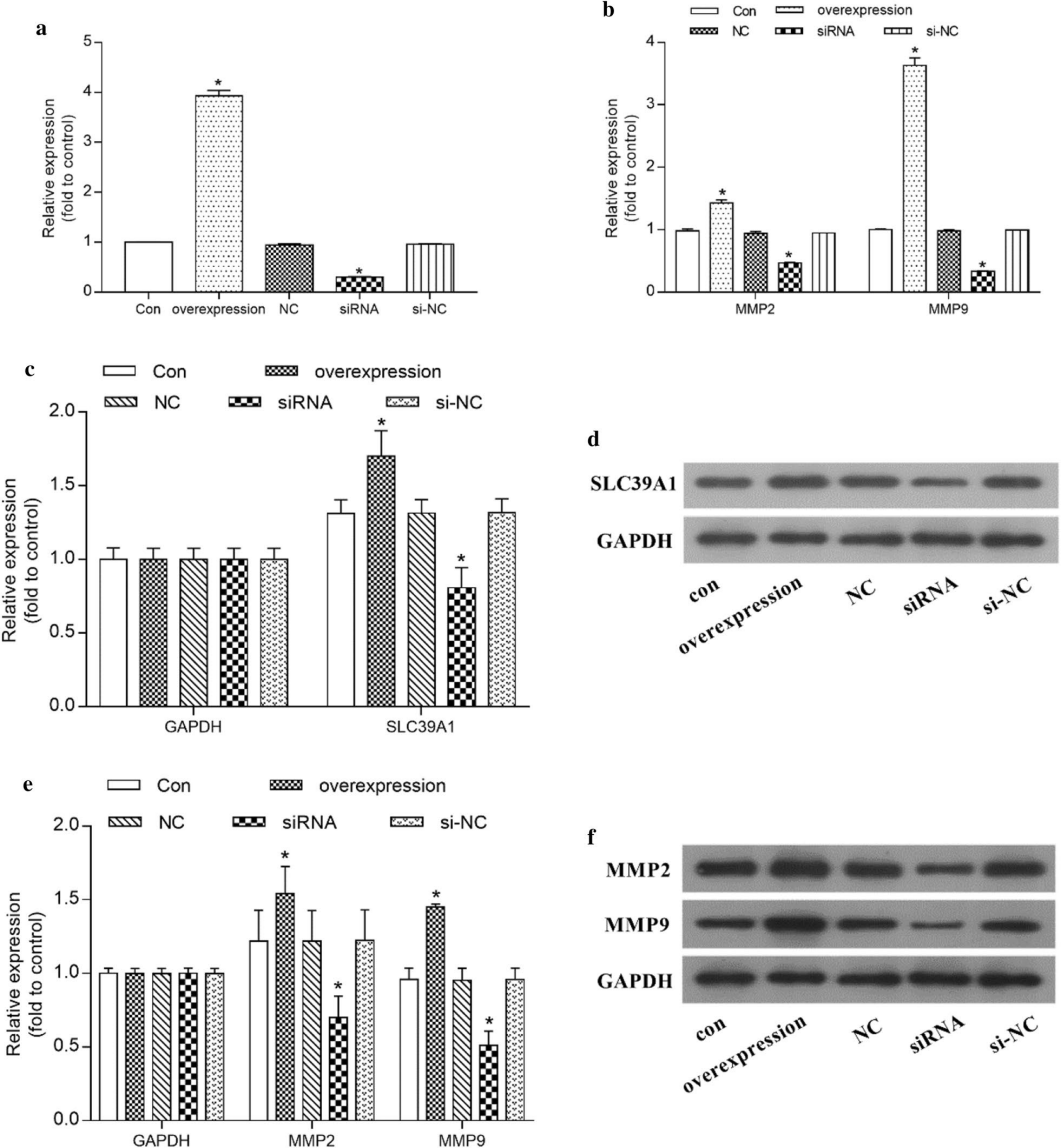
Effect of SLC39A1 on MMP2/MMP9 in glioma. **a, b** The expression of SLC39A1 mRNA (**a**) and protein (**b**) in different groups. **c, d** The expression of MMP2/MMP9 mRNA (**c**) and protein (**d**) in different groups. Con, negative control group; overexpression, SLC39A1 overexpression group; NC, plasmid control group; siRNA, SLC39A1 knockdown groups; si-NC, negative control siRNA. *P<0.05

In order to further study the relationship between SLC39A1 and metalloproteinase MMP2/MMP9, we used RT-qPCR and western blot to measure expression of SLC39A1 and MMP2/MMP9 mRNA and protein in the different groups. As shown in Fig. 8a, c, d, expression of SLC39A1 mRNA and protein in the overexpression group was significantly increased compared with the Con group (P < 0.05), while that in the siRNA group it was significantly decreased (P < 0.05). As shown in Fig. 8b, e, f, expression of MMP2 and MMP9 mRNA and protein in the overexpression group was significantly increased compared with the Con group (P < 0.05), while in the siRNA group it was significantly decreased (P < 0.05).

### SLC39A1 is associated with immune infiltration of gliomas

The infiltration of immune cells in tumors is closely related to clinical results. Therefore, we used the ESTIMATE algorithm to estimate tumor stroma cell and immune cell infiltration scores, and calculated the correlation between them and SLC39A1 expression.

**Fig. 9:**
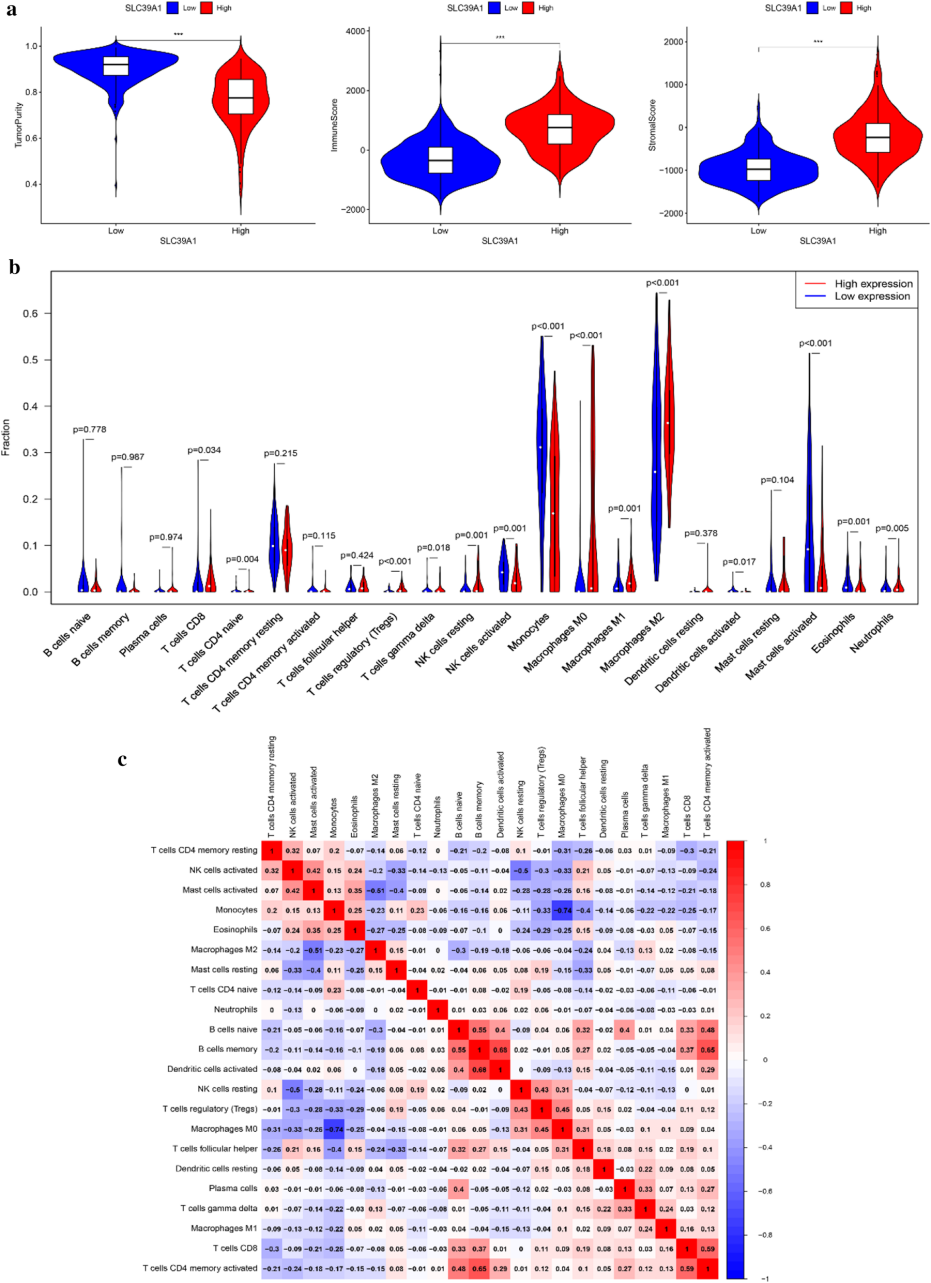
Correlation between the expression of SLC39A1 and immune infiltration of patient with glioma. **a** Correlations between SLC39A1 expression and immune, stromal, and tumor purity scores (from ESTIMATE). **b** The varied proportions of 22 subtypes of immune cells in high and low SLC39A1 expression groups in glioma samples. **c** Heatmap of 22 immune infiltration cells in glioma samples

The results showed that the SLC39A1 expression was significantly and positively correlated with ImmuneScore and StromalScore, and significantly and negatively correlated with TumorPurity (Fig. 9a). We also used the CIBERSORT algorithm to analyze the expression levels of 22 immune cell subgroups, and evaluated their correlation with SLC39A1 expression. The results showed that T cells regulatory (T regs), T cells gamma delta, Macrophages M0, Macrophages M1, Macrophages M2 and Eosinophils were significantly positively correlated with SLC39A1 expression (P < 0.05), among which Macrophages M2 had the strongest correlation; T cells CD4 + naïve NK cells activate, Monocytes, and Mast cells activated were significantly and negatively correlated with SLC39A1 expression, among which NK cells activate had the strongest correlation (Fig. 9b, Table 1). In addition, we also evaluated the possible correlation between 22 immune cells, and the heatmap showed that the ratios of the different tumor infiltration immune cell subgroups were weakly to moderately correlated (Fig. 9c).

**Table 1:** Correlation between SLC39A1 and 22 subtypes of immune cell in glioma samples

Inmune cells	Cor	P
Macrophages M2	0.336	< 0.001
Macrophages M0	0.309	< 0.001
Eosinophils	0.261	< 0.001
T cells regulatory (Tregs)	0.195	< 0.001
T cells gamma delta	0.18	< 0.001
Macrophages M1	0.093	0.013
T cells CD4 memory activated	0.081	0.032
Dendritic cells activated	0.067	0.076
NK cells resting	0.053	0.16
T cells CD4 memory resting	0.051	0.18
Neutrophils	0.022	0.564
T cells CD8	−0.033	0.384
Plasma cells	−0.039	0.305
Dendritic cells resting	−0.049	0.192
B cells naive	−0.094	0.012
B cells memory	−0.101	0.007
Mast cells resting	−0.18	< 0.001
T cells follicular helper	−0.181	< 0.001
Monocytes	−0.193	< 0.001
Mast cells activated	−0.211	< 0.001
T cells CD4 naive	−0.24	< 0.001
NK cells activated	−0.268	< 0.001

## Discussion

Glioma is one of the most common and deadly primary brain tumors [17, 18]. Despite years of unremitting efforts by neurosurgery researchers, the prognosis of glioma patients is still unsatisfactory [19]. Therefore, it is of great significance to find biomarkers that can improve its prognosis.

In recent years, the role of plasma membrane transport proteins in cancer has received increasing attention [20, 21]. Some transport proteins for essential nutrients are up-regulated in cancer and act as tumor promoters or inhibitors [22]. SLC39A1, also known as ZIP1, is a member of the plasma membrane transport protein SLC39A family. Studies have shown that it can affect the citrate metabolism of prostate cancer patients, thereby inhibiting the progression of prostate cancer [15, 22]. However, its role in the progression and prognosis of gliomas is still unclear. In this study, K-M survival analysis, univariate Cox analysis, multivariate Cox analysis, ROC curve analysis, and other methods are employed, using the TCGA, GEO and CCGA databases, to show that SLC39A1 independently indicated poor prognosis in patients with gliomas. Furthermore, correlation analysis has showed that the expression level of SLC39A1 is significantly correlated with PRS type, histology, grade, age, chemo status, IDH mutation status, and 1p19q codeletion status (Fig. 4), suggesting that SLC39A1 may be related to glioma progression.

Zinc is one of the essential trace elements for the human body, and plays an important role in its biological processes, but high concentrations are toxic [23]. It is also a key component of many important enzymes, such as matrix metalloproteinase (MMP) and carbonic anhydrase [24], which are related to tumor proliferation and metastasis [25–28]. Studies have shown that MMP2\MMP9 are zinc-dependent proteolytic enzymes involved in the remodeling and degradation of extracellular matrix (ECM) and can enhance the invasion and metastasis of glioma [29–31]. In this study, GO analysis has shown that SLC39A1 is mainly enriched in extracellular matrix organization, and extracellular structure organization, etc.; KEGG analysis shows that SLC39A1 is mainly enriched in ECM-receptor interaction, etc. (Figure 5). In addition, correlation analysis indicates that SLC39A1 is highly correlated with MMP2\MMP9 (Fig. 6), suggesting that the up-regulated expression of SLC39A1 may promote the progression of glioma by increasing the intake of zinc ions and increasing the expression level of MMP2\MMP9. To further confirm this hypothesis, we conducted in vitro experiments. CCK-8 and flow cytometry results show that SLC39A1 promotes proliferation of glioma cells and inhibits their apoptosis; RT-qPCR and western blot results show that SLC39A1 promotes the progression of glioma by increasing the expression level of MMP2\MMP9 (Fig. 8).

Studies have shown that immune cell infiltration of tumors has become one of the important prognostic indicators for many cancers and one of the important factors affecting the tumor microenvironment [32–34]. ECM depletion is an important link in tumor invasion and metastasis [35, 36]. MMP9 and MMP2 are important members of the MMPs family, secreted by neutrophils, macrophages and tumor cells, which participate in the depletion of ECM and play an important role in the vascularization of malignant tumors and the infiltration of tumor cells [37–40]. In this study, GO analysis and KEGG pathway analysis have showed that the main functions and pathways related to SLC39A1 are extracellular matrix organization, extracellular structure organization and ECM-receptor interaction. At the same time, PCR and western blot results showed that SLC39A1 could promote the expression of MMP2\ MMP9. These results suggest that SLC39A1 may be closely related to glioma immunity. In order to prove the relationship between SLC39A1 and tumor immune infiltration, we employed the ESTIMATE algorithm to estimate stroma cell and immune cell infiltration scores in gliomas, and calculated the correlation between them and SLC39A1, finding that SLC39A1 expression was significantly positively correlated with ImmuneScore and StromalScore, and significantly negatively correlated with TumorPurity (Fig. 9a). We also used the CIBERSORT algorithm to analyze the expression levels of 22 immune cell subgroups, and evaluated their correlation with SLC39A1 expression, finding that Macrophages M2, Macrophages M0 and other immune cell subgroups are significantly and positively correlated with its expression, while NK cells Activate, and Monocytes, etc. were significantly negatively correlated with its expression (Fig. 9b, Table 1). Studies have shown that Macrophages M2 can produce a large number of cell factors and interleukins, etc., which can form an immunosuppression microenvironment that promotes growth and invasion of glioma cells. Their infiltration indicates a poor prognosis for advanced gliomas [41, 42]. NK cells, by secreting tumor necrosis factor (TNF) and interferon (IFN), kill susceptible target cellsthereby exerting the cytolytic activity and improving the prognosis of patients with glioma [43]. These results further prove that SLC39A1 affects infiltration of immune cells in the tumor microenvironment and promotes progression of gliomas.

In conclusion, our study shows that SLC39A1 plays an important role in the malignant progression of gliomas. Bioinformatic analysis shows that SLC39A1 expression is up-regulated in glioma tissues. High SLC39A1 expression predicted significantly worse survival. Univariate and multivariate analysis show that SLC39A1 independently indicated poor prognosis in patients with gliomas. The expression of SLC39A1 is significantly correlated with clinical pathological parameters such as Grade, IDH mutation status, and 1p19q codeletion status. In vitro experimental results show that SLC39A1 promotes the proliferation of glioma cells and inhibits their apoptosis, and may be related to MMP2\MMP9 up-regulation. In addition, SLC39A1 may affect the infiltration of immune cells in the glioma microenvironment. These results suggest that SLC39A1 may be a new prognostic biomarker and potential target for treatment of gliomas.

## Supplementary information

**Additional file 1: Figure S1.** SLC39A1 expression is associated with prognosis in patients with glioma. **a, b, c** Kaplan–Meier analyses of patients with glioma (**a**), LGG (**b**) or HGG (**c**) in different expression level of SLC39A1. The red curve represents high expression and the blue curve represents low expression. P<0.05 indicates a significant difference. **d, e, f** ROC (Receiver operator characteristic curve) analysis of SLC39A1 in patients with glioma (**d**), LGG (**e**) or HGG (**f**). *AUC* area under the curve.

**Additional file 2: Figure S2.** Correlation analysis between SLC39A1 expression and clinicopathological parameters using TCGA database. Differential expression of SLC39A1 was significantly related to **a** Age, **b** Grade, **c** IDH mutation status and **d** 1p19q codeletion status. P <0.05 indicates a significant difference.

**Additional file 3: Figure S3** Gene enrichment analysis of SLC39A1 based on TCGA dataset. **a** Gene Ontology analysis. **b** KEGG pathway analysis.

## Data Availability

The datasets used and/or analysed during the current study are available from the corresponding author on reasonable request.
