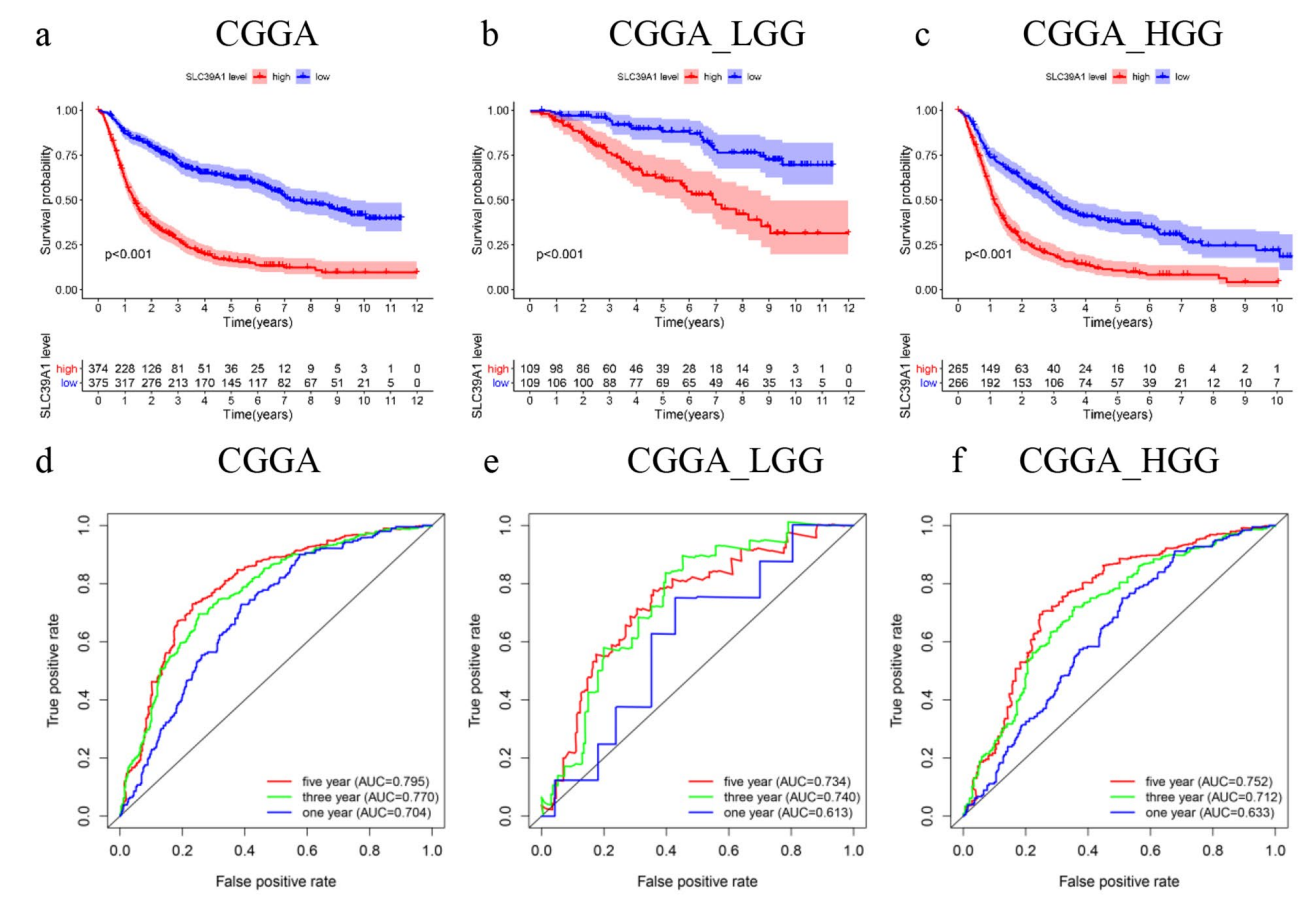# Correction to: SLC39A1 contribute to malignant progression and have clinical prognostic impact in gliomas

**DOI:** 10.1186/s12935-024-03556-2

**Published:** 2024-11-10

**Authors:** Peng Wang, Jingjing Zhang, Shuai He, Boan Xiao, Xiaobin Peng

**Affiliations:** 1 The Fifth Affiliated Hospital of Southern Medical University, Guangzhou, 510900 China; 2 Zhujiang Hospital, Southern Medical University, Guangzhou, 510282 China

**Correction to: Cancer Cell Int (2020) 20:573**

10.1186/s12935-020-01675-0.

In the original publication of this article [1], there was an error in Fig. 2. Due to an oversight in the process of organizing the data, we have used the wrong image in Fig. 2e, f.

The incorrect and correct Fig. 2 are shown in this correction article.

Incorrect Fig. 2.


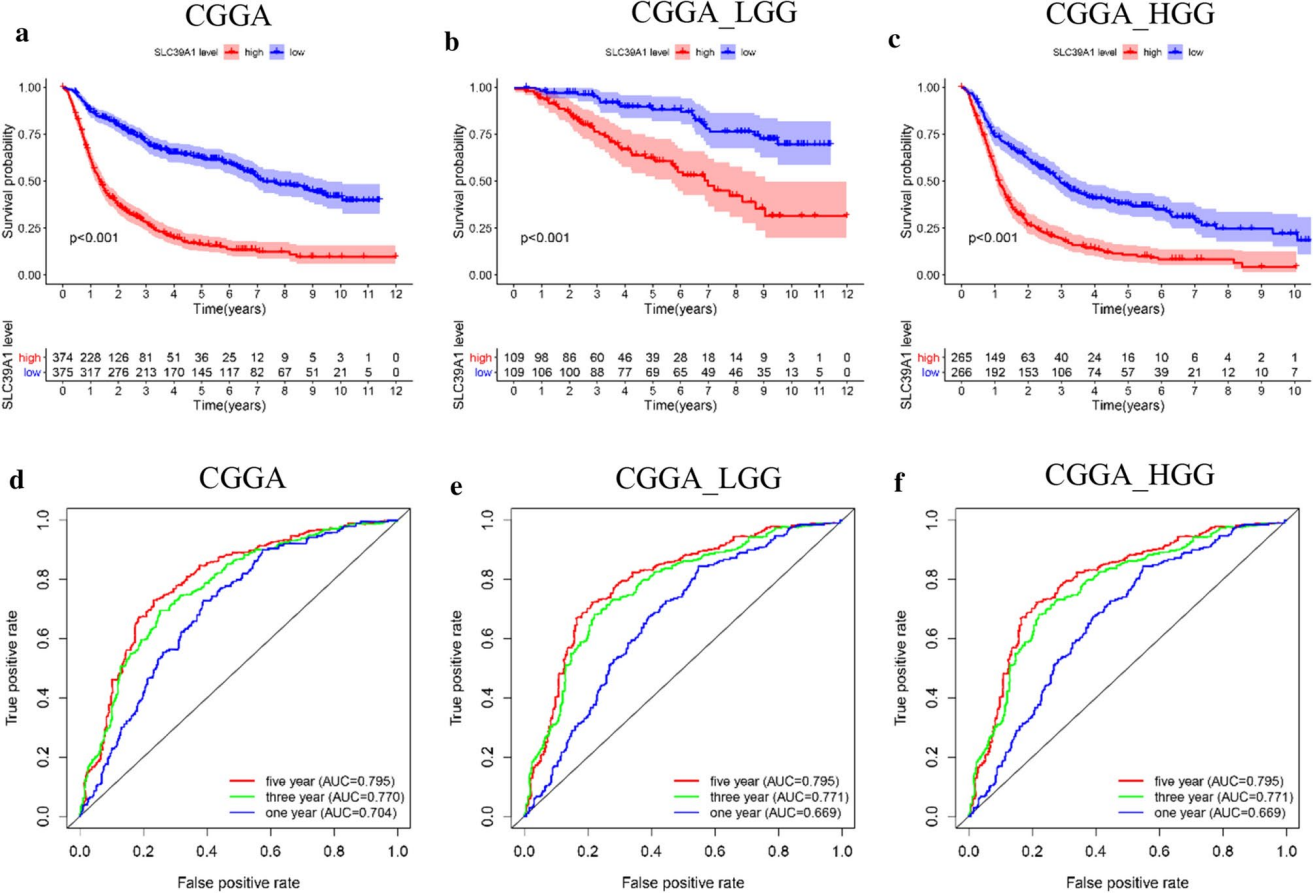


Correct Fig. 2.