# Supplementary figures and images for: SLC39A1 contribute to malignant progression and have clinical prognostic impact in gliomas
Source: Cancer Cell Int. 2020 Nov 27;20:573. doi: 10.1186/s12935-020-01675-0 (PMC7694905; doi:10.1186/s12935-020-01675-0)

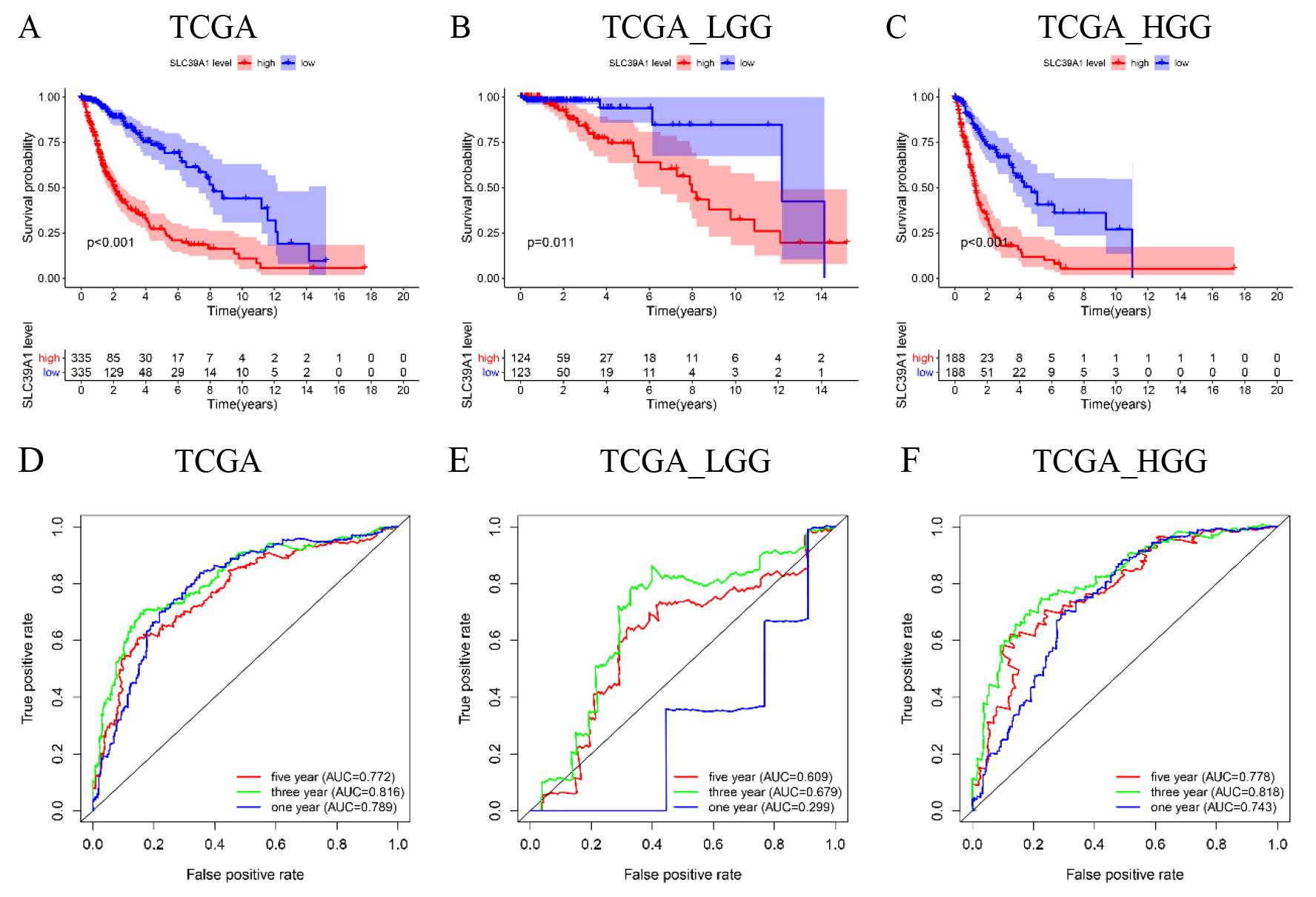

Supplement: Supplementary file 1 — Additional file 1: Figure S1. SLC39A1 expression is associated with prognosis in patients with glioma. a, b, c Kaplan–Meier analyses of patients with glioma (a), LGG (b) or HGG (c) in different expression level of SLC39A1. The red curve represents high expression and the blue curve represents low expression. P<0.05 indicates a significant difference. d, e, f ROC (Receiver operator characteristic curve) analysis of SLC39A1 in patients with glioma (d), LGG (e) or HGG (f). AUC area under the curve. [file 12935_2020_1675_MOESM1_ESM.tif]

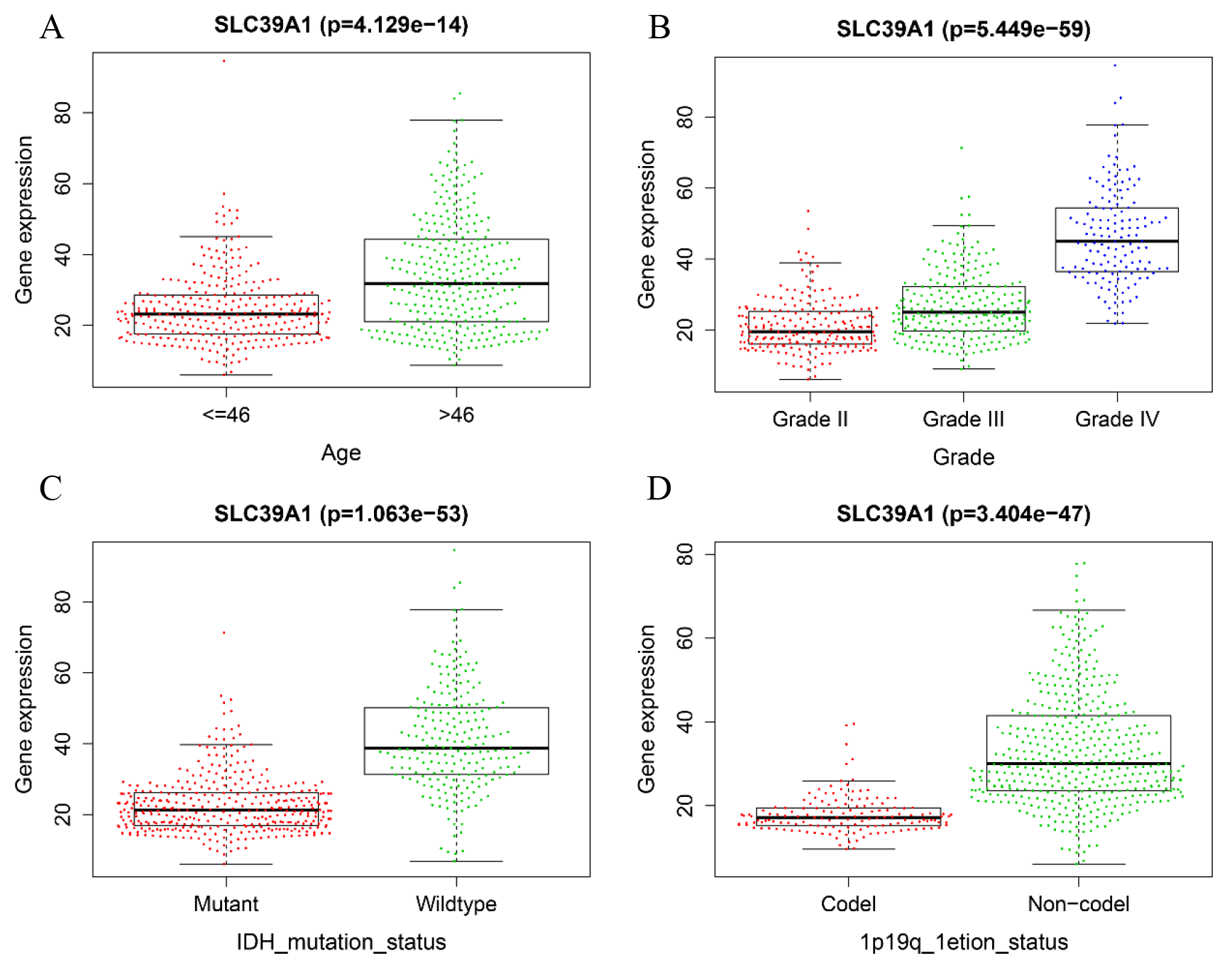

Supplement: Supplementary file 2 — Additional file 2: Figure S2. Correlation analysis between SLC39A1 expression and clinicopathological parameters using TCGA database. Differential expression of SLC39A1 was significantly related to a Age, b Grade, c IDH mutation status and d 1p19q codeletion status. P <0.05 indicates a significant difference. [file 12935_2020_1675_MOESM2_ESM.tif]

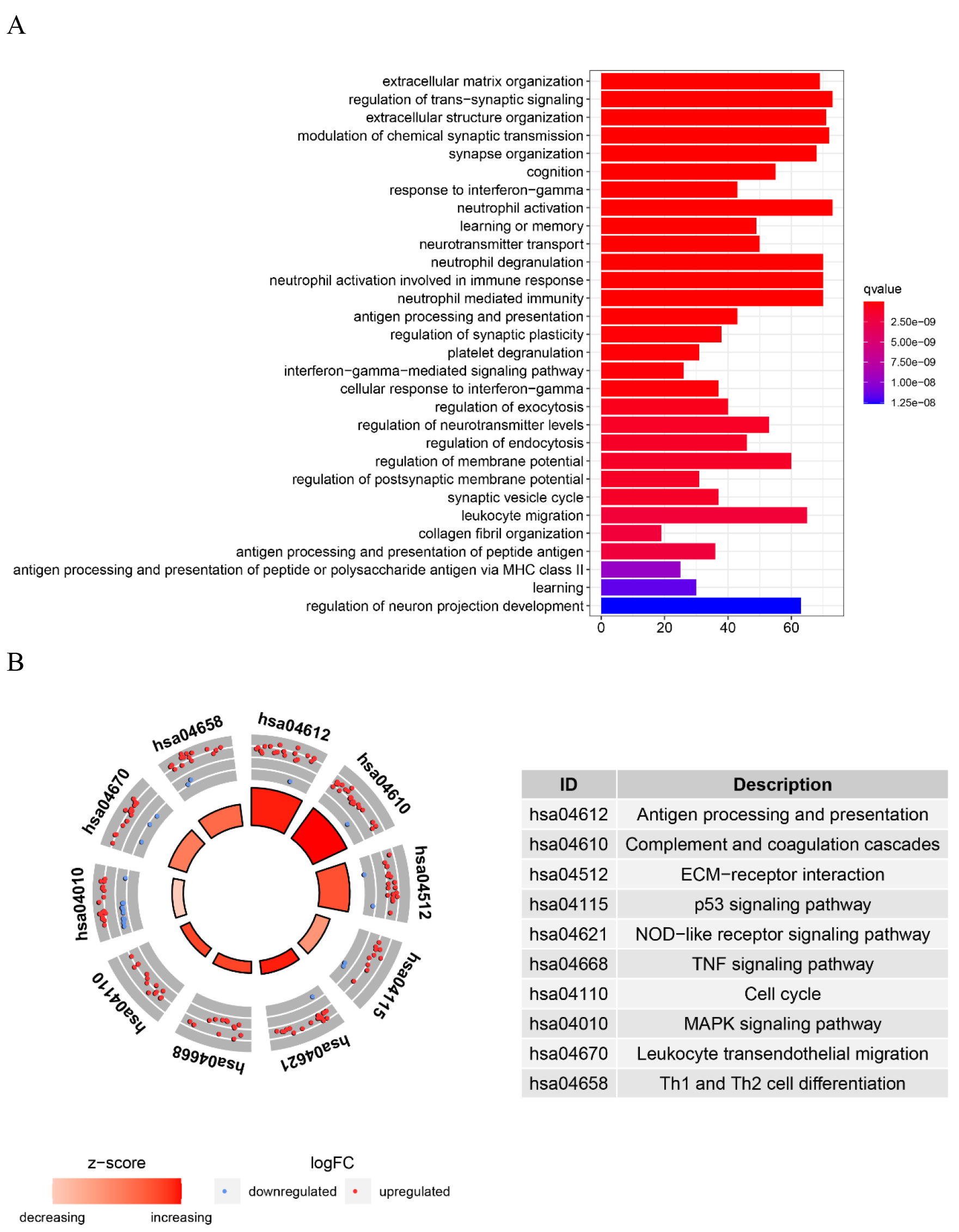

Supplement: Supplementary file 3 — Additional file 3: Figure S3 Gene enrichment analysis of SLC39A1 based on TCGA dataset. a Gene Ontology analysis. b KEGG pathway analysis. [file 12935_2020_1675_MOESM3_ESM.tif]
